# C-reactive protein-triglyceride-glucose index (CTI) as a predictor of chronic liver disease in adults with macrocytic anemia

**DOI:** 10.1038/s41598-026-52488-6

**Published:** 2026-05-09

**Authors:** Wenjing Shang, Jiachen Li, Jinhua Zhuo, Xuewei Zhuang, Jiaxing Li

**Affiliations:** 1 Department of Clinical Laboratory Medicine, Shandong Provincial Third Hospital, Shandong University, Jinan, 250031 China; 2 Zibo Central Hospital, Shandong First Medical University, Zibo, 255036 Shandong China; 3 Shandong Blood Center, Jinan, 250014 Shandong China

**Keywords:** macrocytic anemia, C-reactive protein-triglyceride glucose index, chronic liver disease, CHARLS, longitudinal research, Biomarkers, Diseases, Gastroenterology, Medical research, Risk factors

## Abstract

Macrocytic anemia is frequently associated with liver dysfunction; however, reliable biomarkers for predicting chronic liver disease (CLD) in this population remain limited. This longitudinal study included 693 individuals with macrocytic anemia from the China Health and Retirement Longitudinal Study (CHARLS). Individuals with a mean corpuscular volume (MCV) > 100 fL were defined as having macrocytic anemia. C-reactive protein-triglyceride-glucose index (CTI) was calculated by the formula of CTI = 0.412×Ln(CRP [mg/L]) + Ln(TG [mg/dL] × FPG [mg/dL])/2. Then, the association between CTI and CLD was assessed using three sequentially adjusted Cox regression models, along with restricted cubic splines and subgroup analyses. Model 1 was unadjusted; Model 2 adjusted for demographic characteristics including age, gender, marital status, education, and BMI; Model 3 further adjusted for smoking, drinking, sleep night, and socioeconomic status (SES) based on the Model 2. Key predictors were selected by Least Absolute Shrinkage and Selection Operator (LASSO) to construct a clinical nomogram. We found that a higher CTI was linked to a greater chance of developing liver disease, which remained robust after sequential adjustment for confounders. In Model 3, the hazard ratio (HR) for CTI was 1.607 (95% confidence interval [CI]: 1.085–2.381; *P* = 0.018). Restricted cubic spline analysis confirmed a linear positive relationship between CTI and CLD risk. This link was consistent across different ages, genders, and lifestyles. When we built a prediction tool using CTI and other factors like age and marital status, it showed a fair ability to estimate a person’s risk. CTI may serve as a simple and potentially useful indicator to identify individuals with macrocytic anemia who are at a higher risk of chronic liver disease, helping guide earlier prevention efforts.

**Supplementary Information:**

The online version contains supplementary material available at 10.1038/s41598-026-52488-6.

## Introduction

Chronic liver disease (CLD) represents a major global health challenge, characterized by progressive liver inflammation and fibrosis. Its common symptoms are non-specific, including fatigue and abdominal discomfort, but its systemic effects and long-term consequences are severe, culminating in cirrhosis, liver failure, and hepatocellular carcinoma^1–3^. Notably, the co-occurrence of CLD and macrocytic anemia is frequently observed in clinical practice^4,5^—with both conditions sharing overlapping non-specific symptoms such as fatigue, which may obscure their mutual influence in clinical settings. Macrocytic anemia, a hematologic condition characterized by enlarged red blood cells (typically defined as a mean corpuscular volume (MCV) greater than 100 fL), often manifests with symptoms such as fatigue, weakness, and pallor. There are growing evidences of a bidirectional link between these two disorders: anemia reduces blood oxygen-carrying capacity, causing hepatic hypoxia^6,7^. As a high-metabolism organ, the liver is prone to dysfunction under this oxygen debt; compensatory hepatic hyperperfusion imposes long-term burden, disrupting compensation, promoting fibrosis, and facilitating CLD onset^8^. Furthermore, prolonged administration of specific macrocytic anemia therapies that rely on hepatic metabolism has been suggested to potentially increase hepatic detoxification burden, with a theoretical risk of precipitating drug-induced liver injury (DILI) or worsening underlying hepatic damage^9,10^. Given this intricate link, identifying reliable biomarkers to predict CLD risk in individuals with macrocytic anemia is a critical unmet need in preventive medicine.

In the context of various conditions, including autoimmune and metabolic disorders, metabolic reprogramming occurs in immune cells, inducing proinflammatory or anti-inflammatory effects^11^. The C-reactive protein-triglyceride-glucose index (CTI) is a novel composite biomarker that concurrently captures inflammation (C-reactive protein, CRP) with metabolic dysregulation (triglycerides and glucose). Its advantage lies in its ability to provide a holistic reflection of two key pathophysiological pathways, coupled with the benefits of being easily obtainable and quantitatively robust. This has positioned CTI as a promising tool for risk stratification in various chronic diseases^12^. Since both inflammation and metabolic issues are known to harm the liver and are common in people with anemia, a marker like CTI that captures both at once might be especially powerful. Although a prospective cohort study by Zheng et al. using the CHARLS database has confirmed that elevated CTI is independently associated with an increased risk of incident liver disease in the general middle-aged and older Chinese population^13^, that investigation was restricted to analyses of the general population and did not examine the risk stratification value of CTI in the specific high-risk subgroup with macrocytic anemia. Given that macrocytic anemia itself is linked to elevated liver disease risk, whether CTI retains an independent predictive role and enables more precise liver disease risk stratification in this clinically prevalent high-risk population remains unknown. This key evidence gap represents the primary novelty and focus of the present study.

In this study, we aimed to investigate whether CTI could predict the risk of chronic liver disease in a large, national sample of middle-aged and older Chinese adults with macrocytic anemia. Our goal was to find a simple, blood-based way to identify those at highest risk, so we could potentially intervene earlier.

## Materials and methodology

### Longitudinal study design and population characteristics

**Fig. 1:**
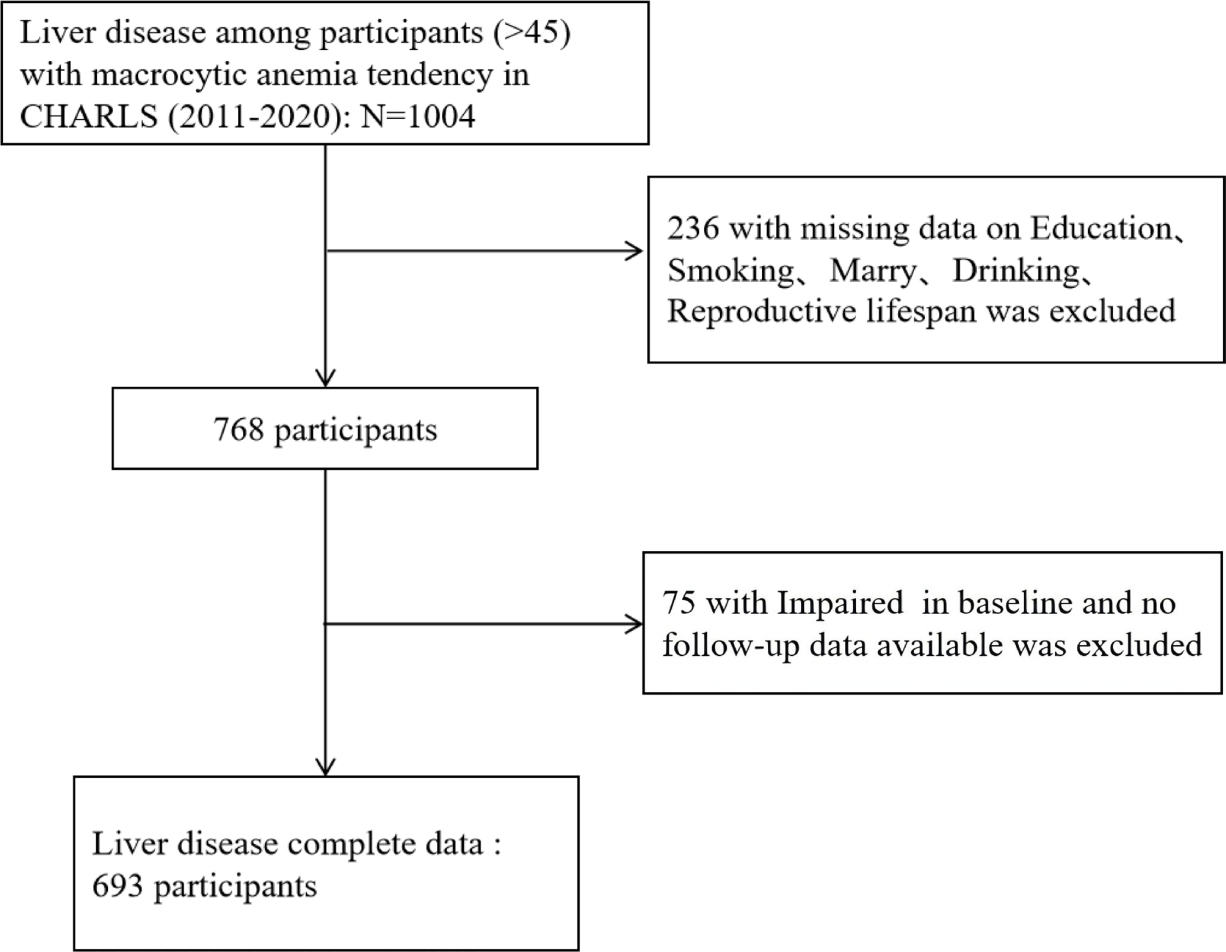
The inclusion and exclusion flowchart of work.

The China Health and Retirement Longitudinal Study (CHARLS) database (https://charls.pku.edu.cn/) provided the demographic information used in this investigation. The organizer, Peking University’s National Development Institute, hopes to further multidisciplinary research on population aging in China by gathering high-quality microdatasets from representative cohorts of people 45 years of age and older. The CHARLS study employed a pilot survey in the provinces of Gansu and Zhejiang in 2008. Beginning in 2011, baseline surveys were carried out every two years. In 28 provinces, the survey included 450 community-village level units and 150 county-level units. Every subject gave written informed permission, and each CHARLS study was authorized by Peking University’s Biomedical Ethics Committee (IRB00001052-11015). Data derived from the CHARLS over the decade 2011–2020 were used to conduct a longitudinal analysis. Participants were restricted to those with macrocytic anemia (MCV > 100 fL). The study comprised 693 middle-aged and elderly participants, who were chosen based on the following selection criteria: (1) age ≥ 45 years; (2) MCV > 100 fL; (3) No baseline CLD; (4) Available follow-up data (Fig. 1).

### Determination of CTI

The exposure factor in our study was CTI. CTI wa*s* computed by the following formula^14^:

$$\:CTI=0.412\times\:Ln\left[CRP\right(mg/L\left)\right]+Ln\left[TG\right(mg/dL)\times\:FPG(mg/dL\left)\right]/2$$

### Definition of outcome

Referring to previous literature^15^, we defined the outcome event as self-reported CLD. Specifically, whether participants had received a doctor’s confirmed diagnosis of liver disease (excluding fatty liver, cancer, and tumors). It is important to acknowledge that defining CLD based on self-reported physician diagnosis constitutes a potential limitation of this study, as it may be subject to recall bias or misclassification of disease status.

### Covariates

To evaluate the influence of possible confounders, many significant covariates were chosen for this investigation. Age, gender (male, female), marital status (married, other), educational attainment (primary education or lower, secondary education and above), body mass index (BMI), smoking status (yes, no), drinking status (yes, no), sleep night (continuous variable), and socioeconomic status (SES: low, middle, high). Smoking and drinking status were defined as current status, while sleep night was defined as the average duration of actual sleep each night over the past month. The SES is calculated by combining educational attainment and family income. Educational attainment is uniformly classified into three levels based on the specific classification of the study: junior high school or below (0 points), high school and vocational training (1 point), and higher education (2 points). The calculation of total family wealth is based on the assessment point of health status, including all wealth components such as real estate, business assets, vehicles, and savings accounts (excluding liabilities). To facilitate cross-cohort comparison, the total family wealth in each study is grouped into quartiles, with scores of 0–3 corresponding to the lowest (first quartile) to the highest (fourth quartile) wealth levels. The total SES score of this study is the sum of educational attainment and total family wealth scores, and is divided into three grades: low (0 points), medium (1–2 points), and high (≥ 3 points)^16^.

### Statistical analysis

The normality of continuous variables was assessed using the Shapiro-Wilk test. Data were summarized as mean ± standard deviation for normally distributed variables, with group comparisons made by the independent samples t-test. For non-normally distributed variables, data were presented as median (IQR) and compared using the Kruskal-Wallis test. Categorical variables were expressed as n (%) and analyzed with the chi-square test.

Longitudinal analyses were carried out using the Cox proportional hazards regression model (survey package, version 4.2-2)^17^. The association between exposure factors and outcomes was examined through three progressively adjusted models: Model 1 was an unadjusted crude model; Model 2 incorporated adjustments for demographic characteristics (including age, gender, marital status, education, and BMI); Model 3 further adjusted for smoking, drinking, sleep night, and SES based on Model 2. Restricted cubic splines (RCS) analysis (rms package, version 6.8-0^18^ was employed to assess the dose-response relationship between CTI and CLD. In the Cox regression model, the Schoenfeld residuals test confirmed that the core variables satisfied the proportional hazards assumption (Table S1).

Subgroup analyses were conducted using stratification based on the covariates from Model 3. Stratified Cox regression was used to evaluate the modifying effect of different population characteristics on effect sizes. Furthermore, a clinical prediction nomogram (regplot package, version 1.1)^19^ was constructed based on the Least Absolute Shrinkage and Selection Operator (LASSO) algorithm (glmnet package, version 4.1.10). By integrating key predictive variables including gender and education level, this nomogram enables intuitive quantitative prediction of individual patients’ survival probabilities, facilitating rapid clinical assessment of prognostic differences among populations with distinct characteristics. In this research, missing data were handled by complete-case analysis. Participants with missing values in key baseline or outcome variables were excluded from the final models. All statistical analyses were performed using R software (version 4.4.3), and a two-tailed *P*-value of less than 0.05 was considered statistically significant.

## Results

### Baseline characteristics

This study enrolled 693 participants with macrocytic anemia (MCV > 100 fL), of whom 63 developed CLD during follow-up (Table 1). Among the baseline characteristics, marital status and nightly sleep duration significantly differed between the without liver disease and with liver disease (CLD) groups (*P* = 0.006 and *P* = 0.005, respectively). Notably, the CLD group exhibited a higher proportion of married individuals (93.7% vs. 80.3%) and a reduced median sleep duration [6.00 (4.00, 7.00) vs. 6.00 (5.00, 8.00) hours] relative to the without liver disease group. Additionally, CTI showed a borderline difference, with a higher median in the CLD group [4.82 (4.33, 5.30) vs. 4.61 (4.30, 5.05), *P* = 0.065]. The age, education, BMI, smoking, drinking, and SES category did not exhibit statistically significant differences. In terms of gender distribution, participants who developed incident CLD had a substantially higher proportion of males compared with those without liver disease (73.0% vs. 65.6%), corresponding to a male-to-female ratio of approximately 2.7:1 in the CLD group.

**Table 1:** Baseline data.

Variable	Overall	Without liver disease	With liver disease	*P*-value
*n*	693	630	63	
CTI (median [IQR])	4.62 [4.30, 5.08]	4.61 [4.29, 5.05]	4.82 [4.33, 5.30]	0.065
Age (median [IQR])	60.00 [54.00, 68.00]	60.00 [54.00, 68.00]	60.00 [56.00, 68.00]	0.546
Gender (%)				0.265
Female	234 (33.8)	217 (34.4)	17 (27.0)	
Male	459 (66.2)	413 (65.6)	46 (73.0)	
Marry (%)				0.006
Married	565 (81.5)	506 (80.3)	59 (93.7)	
Other	128 (18.5)	124 (19.7)	4 (6.3)	
Education (%)				0.285
Primary education or lower	648 (93.5)	591 (93.8)	57 (90.5)	
Secondary education and above	45 (6.5)	39 (6.2)	6 (9.5)	
BMI (mean (SD))	25.97 (3.85)	26.29 (3.82)	22.73 (4.16)	0.77
Smoking (%)				0.509
NO	353 (50.9)	318 (50.5)	35 (55.6)	
YES	340 (49.1)	312 (49.5)	28 (44.4)	
Drinking (%)				0.692
NO	380 (54.8)	347 (55.1)	33 (52.4)	
YES	313 (45.2)	283 (44.9)	30 (47.6)	
sleep night (median [IQR])	6.00 [5.00, 8.00]	6.00 [5.00, 8.00]	6.00 [4.00, 7.00]	0.005
SES category (%)				0.528
Low	165 (23.8)	148 (23.5)	17 (27.0)	
Middle	343 (49.5)	316 (50.2)	27 (42.9)	
High	185 (26.7)	166 (26.3)	19 (30.2)	

### Higher CTI level as an Independent Predictor of CLD

**Fig. 2:**
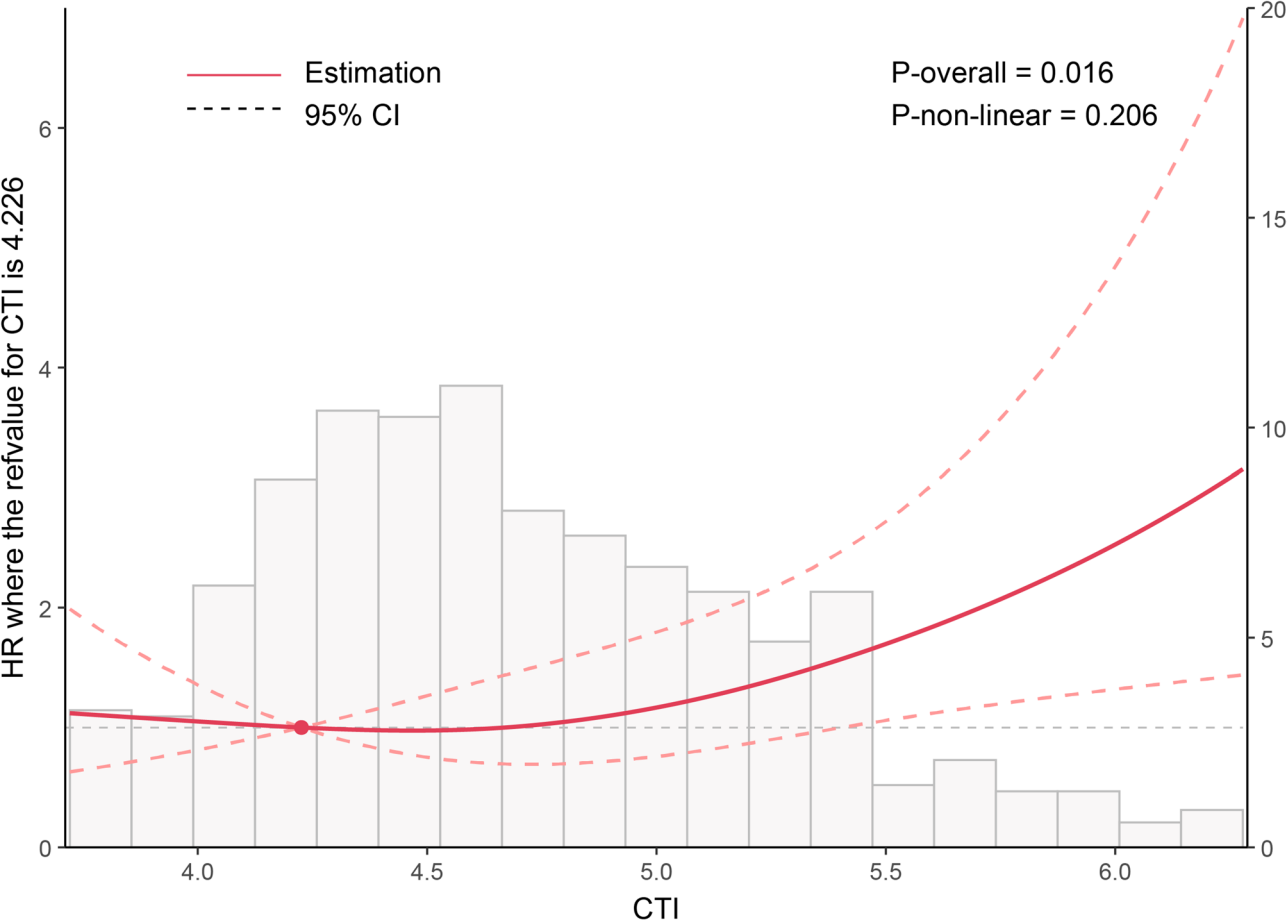
Restricted cubic spline (RCS) model was used to examine the dose-response relationship between C-reactive protein-triglyceride glucose index (CTI) and chronic liver disease.

To delineate the risk of CLD development in normal individuals, Cox regression analysis was conducted to assess the association between CTI and subsequent CLD onset (Table 2**)**. The results demonstrated that CTI was significantly positively correlated with the risk of CLD. In Model 1 (the unadjusted crude model), CTI was associated with a hazard ratio (HR) of 1.597 (95% confidence interval [CI]: 1.085–2.351, *P* = 0.018). After adjusting for demographic characteristics (age, gender, marital status, etc.) in Model 2, the HR was 1.617 (95% CI: 1.072–2.441, *P* = 0.022). In Model 3, which further adjusted for lifestyle factors (smoking, drinking, etc.) based on Model 2, the HR was 1.607 (95% CI: 1.085–2.381, *P* = 0.018). This hazardous effect remained statistically significant after stepwise adjustment for different confounding factors, indicating that a higher CTI level is an independent risk factor for CLD. The dose-response relationship of Model 3 was examined using the RCS model (Fig. 2**)**. There was a significant dose-response relationship between CTI and CLD (*P*-overall < 0.05), and a positive linear trend was observed, indicating that the risk of CLD increased with the increase of CTI.

**Table 2:** Cox regression model.

	Model 1		Model 2			Model3		
	HR(95% CI)	*P*	HR(95% CI)		*P*	HR(95% CI)		*P*
CTI	1.597(1.085–2.351)	0.018	1.617(1.072–2.441)		0.022	1.607(1.085–2.381)		0.018

CTI: C-reactive protein-Triglyceride-glucose Index; HR: Hazard Ratio; CI: Confidence Interval.

### Subgroup analysis

**Fig. 3:**
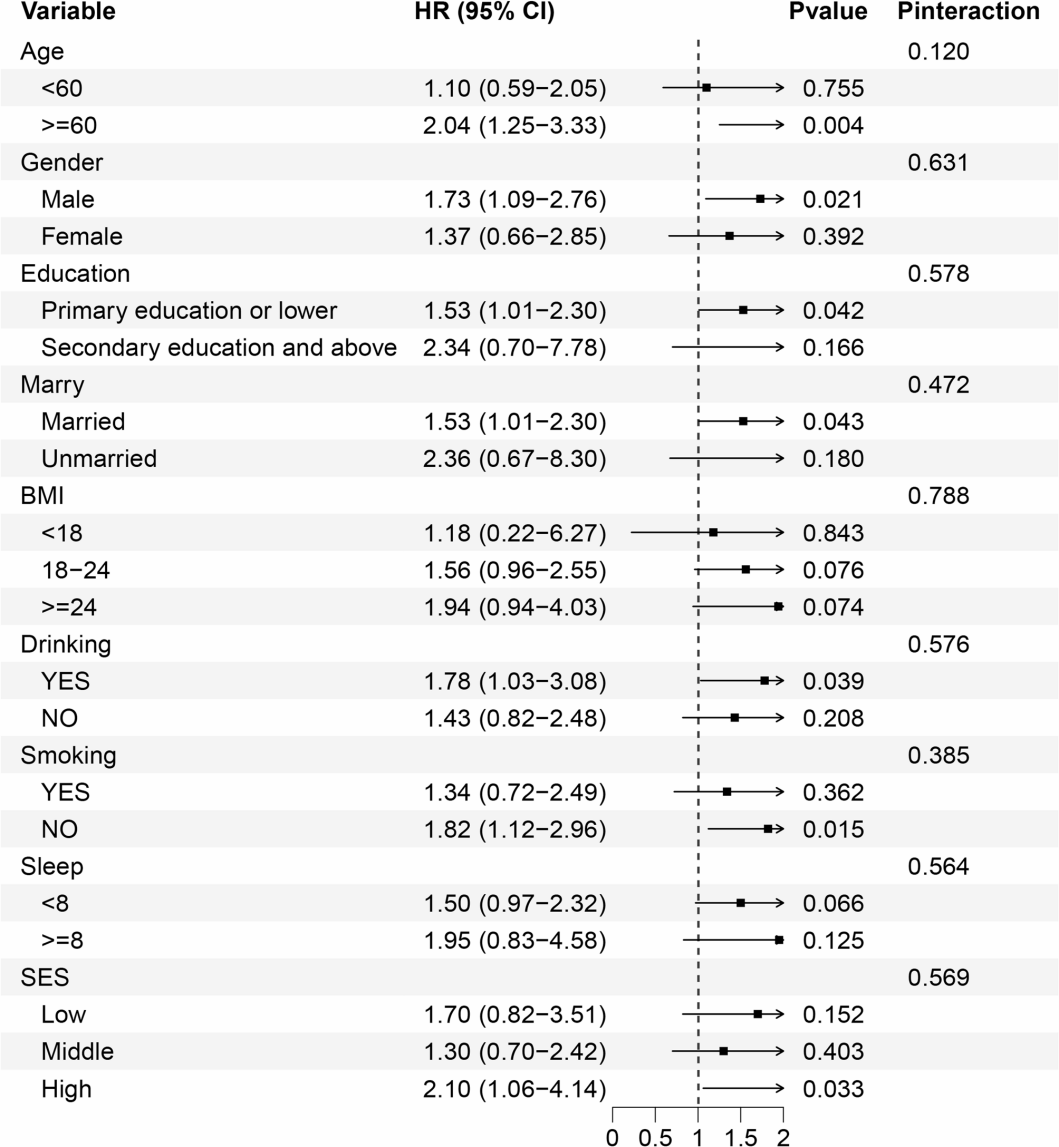
Subgroup analysis.

To further explore the stability of the association between CTI and CLD risk across different populations, subgroup analyses were conducted (Fig. 3). Results showed that CTI was significantly associated with an increased risk of CLD in subgroups including those aged ≥ 60 years (HR = 2.04, 95% CI: 1.25–3.33, *P* = 0.004), males (HR = 2.04, 95% CI: 1.25–3.33, *P* = 0.004), individuals with primary education or lower (HR = 2.53, 95% CI: 1.07-6.00, *P* = 0.036), married individuals (HR = 1.53, 95% CI: 1.01–2.30, *P* = 0.043), and those with high SES (HR = 2.10, 95% CI: 1.06–4.24, *P* = 0.033). Additionally, all interaction terms were not statistically significant, suggesting the association between CTI and CLD risk is consistent across subgroups and not modified by these covariates.

### LASSO regression analysis and construction of the nomogram

**Fig. 4:**
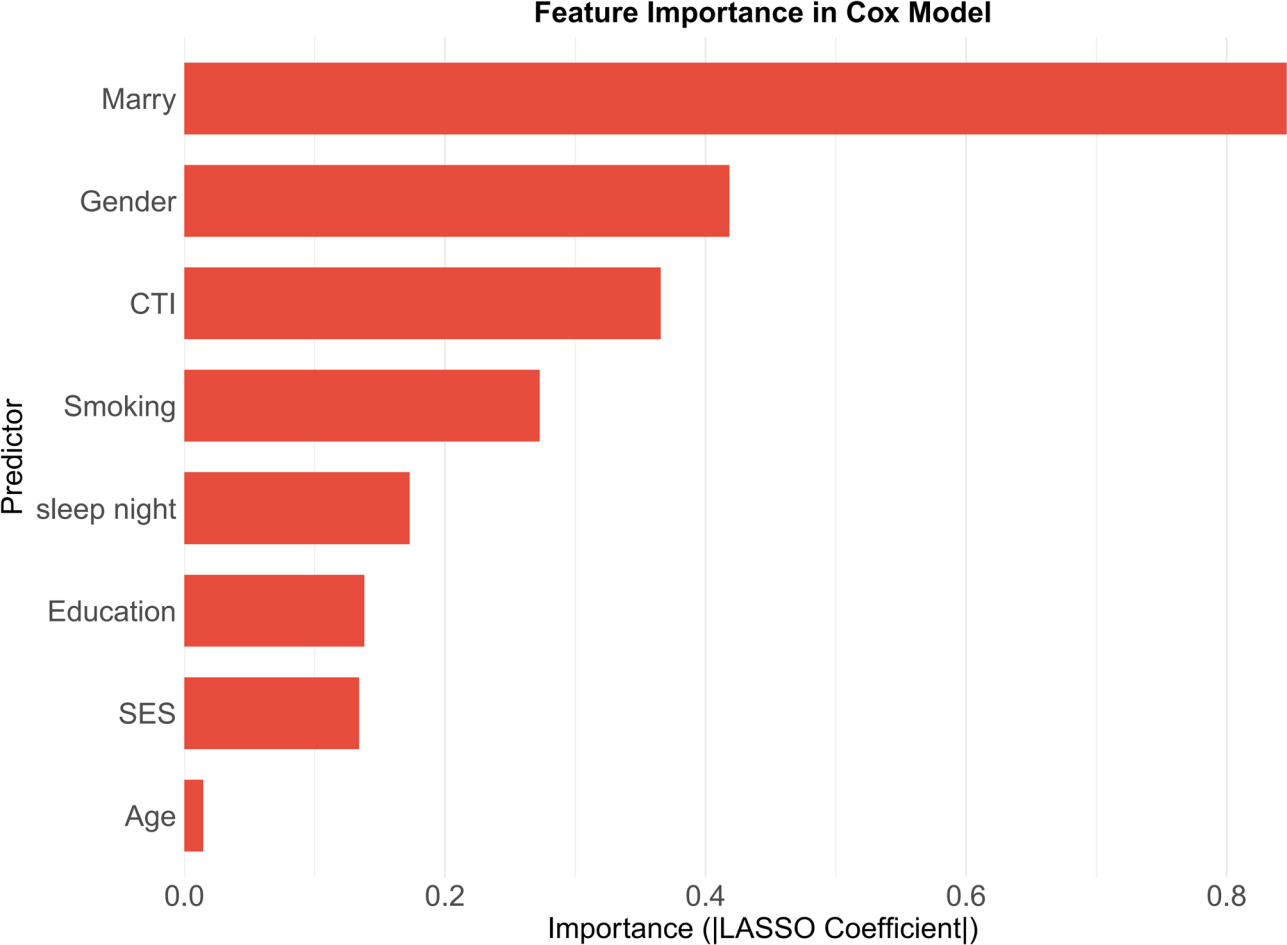
Least absolute shrinkage and selection operator (LASSO) regression was used to screen important variables.

**Fig. 5:**
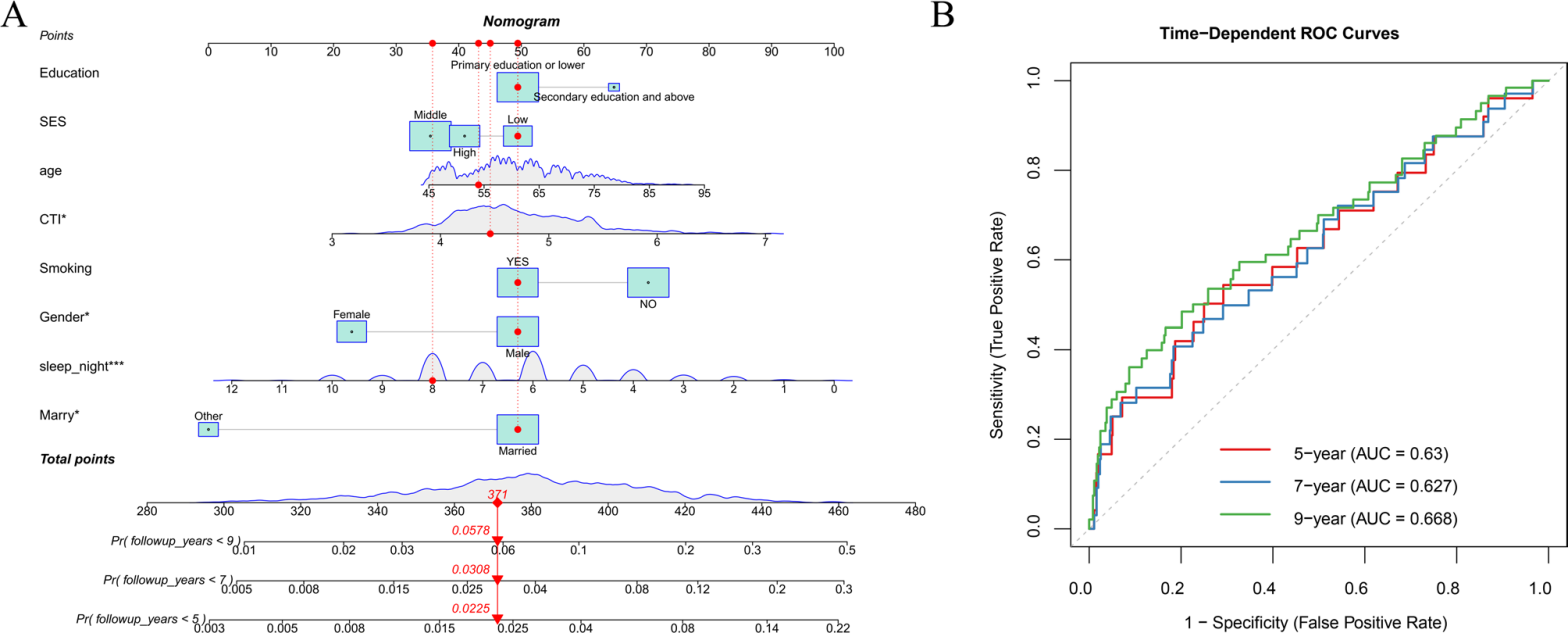
Nomogram and receiver operating characteristic (ROC) curve.

The LASSO model was used to evaluate the relative importance of the selected variables on the risk of CLD. As shown in Fig. 4, CTI was found to be the risk predictor of CLD. The nomogram model integrated the key predictors (marital status, gender, CTI, smoking status, sleep night, education, SES and age), and quantified the individual risk of CLD events in 5, 7, and 9 years through a multivariate scoring system. The model achieves individualized risk assessment through intuitive scale transformation. Each predictor variable was quantified with the use of a point-transformation system, with each variable assigned a point value of 0 to 100 and a total score corresponding to a probability of the risk of CLD. Time-dependent ROC curves confirmed the nomogram’s ability to stratify CLD risk at 5, 7, and 9 years in this high-risk population, with respective area under the curve (AUC) value of 0.63, 0.627, and 0.668. These values indicated a moderate discriminative ability of the model in this high-risk population. While the predictive performance was modest, the results suggest a slight improvement in discriminative ability with longer follow-up durations (Fig. 5).

## Discussion

In this study, we conducted a longitudinal cohort analysis to investigate, for the first time, the value of the CTI in predicting CLD risk in middle-aged and elderly individuals with macrocytic anemia. Our primary finding is that a higher CTI level was independently associated with an increased risk of CLD in this population, and this significant positive association remained stable even after multivariable adjustments. Furthermore, dose-response analysis showed a linear increase in CLD risk with increasing CTI levels, suggesting its potential role as a continuous predictive biomarker. This finding extends the application of CTI from the previously established fields of cardiometabolic diseases to risk prediction for liver disease in this specific high-risk group^20^. The underlying mechanism likely involves the dual pathological processes (inflammation and metabolism) integrated by CTI. On one hand, persistent low-grade inflammation (as reflected by CRP) can directly activate hepatic stellate cells and drive liver fibrosis progression^21,22^. On the other hand, insulin resistance and lipid metabolism disorders, reflected by the TyG index, contribute to abnormal fat deposition in hepatocytes and oxidative stress, thereby initiating and accelerating liver injury^23,24^. In individuals with macrocytic anemia, who may already have underlying metabolic dysregulation due to nutritional deficiencies or compromised hepatic functional reserve—higher CTI levels were associated with a greater superimposed pathophysiological burden, which may help identify those at the highest risk of progressing to CLD.

Although CTI is a relatively new concept, research specifically exploring its application value in liver diseases has emerged in recent years, and the preliminary results are encouraging. A large cross-sectional study based on the National Health and Nutrition Examination Survey (NHANES) database systematically evaluated the relationship between CTI and non-alcoholic fatty liver disease (NAFLD)/liver fibrosis^25^. The study found that there was a significant positive linear correlation between CTI levels and NAFLD/liver fibrosis. More importantly, when comparing diagnostic efficacy, the AUC of CTI for predicting NAFLD and liver fibrosis reached 0.756 (95% CI: 0.740, 0.772) and 0.702 (95% CI: 0.673, 0.731), respectively, which were significantly higher than TyG, hs-CRP, FIB-4, ALT, and AST (*P* < 0.001). Another study also pointed out that CTI was positively correlated with the controlled attenuation parameters (CAP) of American adults, indicating its potential as a reliable indicator for NAFLD^26^. In summary, these research results suggest that CTI performs well in assessing the risk of liver diseases, but further clinical validation is needed to confirm its applicability in routine diagnosis and treatment.

Beyond the core indicator of CTI, our findings highlight that its association with CLD risk is particularly pronounced within specific demographic subgroups, offering insights into potential effect modifiers. The significantly elevated hazard ratios observed in individuals aged ≥ 60 years may be attributable to age-related declines in hepatic regenerative capacity and the increased prevalence of comorbid metabolic conditions, which could amplify the detrimental impact of chronic inflammation and insulin resistance encapsulated by CTI^27,28^. The stronger association in males aligns with established sex disparities in CLD epidemiology, potentially mediated by higher exposure to traditional risk factors such as alcohol use and visceral adiposity, as well as the protective role of estrogen in females^29,30^. The marked risk in participants with primary education or lower suggests that socioeconomic determinants of health, including limited health literacy, dietary patterns, and access to preventive care, may critically intersect with the biological pathway indicated by CTI, creating a synergistic risk environment^31–33^. While the association was significant in married individuals and those with high SES, the lack of significant interaction terms suggests CTI remains a relevant risk indicator across these strata. Collectively, these subgroup analyses underscore that the risk prediction value of CTI is embedded within a complex socio-biological context. They indicate that individuals who are older, male, or from disadvantaged educational backgrounds may represent priority subpopulations for intensified screening when CTI levels are elevated.

A key strength and novelty of our study is the first systematic evaluation and validation of the novel composite biomarker CTI for predicting CLD in a well-defined high-risk population (individuals with macrocytic anemia). However, this study has several limitations: first, our nomogram model has not yet been externally validated in an independent cohort or with more recent datasets, its generalizability needs to be confirmed by future studies. Second, residual confounding may still exist despite multivariable adjustment, as unmeasured factors such as detailed dietary habits, alcohol intake, physical activity, or genetic predisposition were not fully accounted for. Third, while our statistical analyses confirmed a robust CTI-CLD association, the precise biological mechanisms involved, such as how the interaction between CTI and macrocytic anemia specifically impacts hepatocyte function, require further elucidation through basic cellular or animal experiments. Fourth, the outcome (physician-diagnosed CLD) was self-reported, rendering it susceptible to misclassification bias. Fifth, the study population was restricted to Chinese adults aged 45 years and older, which may limit the generalizability of findings to other age, ethnic, or geographic groups. Sixth, due to the constraints of the CHARLS database, comprehensive information on medications associated with macrocytic anemia or other therapeutic drugs was not systematically collected. Therefore, these factors could not be adjusted for in the analysis, which may introduce residual confounding. Seventh, this study only included individuals with macrocytic anemia, a group with a higher risk of liver diseases themselves, and thus there was a selection bias. Additionally, the CHARLS database lacks sensitive liver markers such as ALT and GGT, making it impossible to exclude individuals with latent liver diseases at the baseline. It is difficult to strictly distinguish between newly developed and existing liver diseases, and the results are more inclined to identify the risk of existing or subclinical liver damage. Finally, the association between CTI and CLD, as indicated by a hazard ratio (HR ≈ 1.6), was indeed of “moderate” intensity, and the confidence interval was relatively wide. This finding directly reflected the relatively limited number of CLD events in this study (*n* = 63), which restricted the statistical power. This limitation may have made the results less robust than those from studies based on larger sample sizes and more events. Therefore, future large-scale prospective cohort studies are needed, with the recruitment of more participants and the collection of longer-term follow-up data to obtain more events, in order to verify and more accurately quantify this association.

## Conclusion

In summary, CTI is a simple, inexpensive blood test that could help identify people with macrocytic anemia who are at a higher risk of chronic liver disease. While more work is needed to refine the prediction, our findings support the potential value of CTI as a preliminary risk stratification tool in this vulnerable population.

## Electronic Supplementary Material

Below is the link to the electronic supplementary material.

Supplementary Material 1

## Data Availability

The datasets generated or analyzed during this study are available in the CHARLS database (https://charls.pku.edu.cn/).

## Abbreviations

CLD: Chronic liver disease
DILI: Drug-induced liver injury
CTI: C-reactive protein-triglyceride-glucose index
CRP: C-reactive protein
CHARLS: China health and retirement longitudinal study
RCS: Restricted cubic splines
MCV: Mean corpuscular volume
BMI: Body mass index
LASSO: Least absolute shrinkage and selection operator
AUC: Area under the curve
CI: Confidence interval
HR: Hazard ratio
